# Periprosthetic hip infections in a Swedish regional hospital between 2012 and 2018: is there a relationship between *Cutibacterium acnes* infections and uncemented prostheses?

**DOI:** 10.5194/jbji-6-219-2021

**Published:** 2021-06-04

**Authors:** Urban Hedlundh, Michail Zacharatos, Jonas Magnusson, Magnus Gottlander, Johanna Karlsson

**Affiliations:** 1 Department of Orthopedic Surgery, Uddevalla Hospital, SE 451 80, Uddevalla, Sweden; 2 Department of Infectious Diseases, NU Hospital Group, Trollhättan/Uddevalla, Sweden; 3 Department of Infectious Diseases, Institute of Biomedicine, Sahlgrenska Academy, University of Gothenburg, Gothenburg, Sweden

## Abstract

The purpose of this study was to evaluate patients requiring in-patient care due to a periprosthetic joint infection (PJI), with respect to bacterial agents, surgical treatment, antibiotics, and outcome. We retrospectively identified all infected total hip arthroplasties (THAs) in a Swedish regional hospital during a 7-year period (2012–2018) and reviewed medical records and microbiological data. A total of 89 infected THAs in 87 patients were identified. Standardized treatment with debridement with retention of the implant and antibiotics (DAIR) was initially performed in 53 cases (60 %), one or two stage revisions in 33 cases (37 %), and an immediate Girdlestone in 3 cases (3 %). Infection eradication was seen in 77 PJIs (87 %) in addition to six patients (7 %) ending up with a permanent but uninfected Girdlestone. All six patients with manifest failures were infected with *Staphylococcus aureus*, two of which were also polymicrobial. *Cutibacterium acnes* was found in 18 of 89 patients (16 %) distributed in 15 uncemented implants but only in 3 hybrids and cemented arthroplasties, while remaining pathogens were equally distributed in uncemented THAs (n=31) and THAs with at least one cemented component (n=40; p=0.003). Eradication was achieved in all 18 patients when *Cutibacterium acnes* was the only culture (n=14) or clearly dominant among positive cultures (n=4). DAIR was successful in selected postoperative infections up to 6 months after hip replacement. *Cutibacterium acnes* infections in hip arthroplasty may be underdiagnosed. Cemented components in THAs seem to protect from colonization with *Cutibacterium acnes*.

## 1 Introduction

Periprosthetic joint infection (PJI) is a feared and serious complication of prosthetic surgery, which causes great suffering for the individual and requires major healthcare resources. The incidence in primary interventions is 0.5 %–2 %, higher for revision procedures (Engesaeter et al., 2011), and seems to be increasing both worldwide (Kurtz et al., 2012) and in Scandinavia (Dale et al., 2012). Antibiotic treatment alone is insufficient to eradicate and cure infection (Sendi et al., 2017; Zimmerli et al., 2004). Complete exchange of implants, particularly when performed as a two-stage procedure, results in substantial morbidity, loss of ambulation, and decreased quality of life (Leonard et al., 2014). Debridement, antibiotics, and implant retention (DAIR) of well fixated implants has gained increased popularity in an international context due to both increased cure rates and patient preference. Diverse selection criteria, various treatment protocols, insufficient detection of causative bacterial agents, limited surgical accuracy, and inconsistent follow-up significantly limits comparisons between studies (Tsang et al., 2017).

The most common contaminating organism in PJI is *Staphylococcus aureus* followed by coagulase negative staphylococci (CoNS), streptococci, *Escherichia coli*, *Enterococcus* species, and in recent years *Cutibacterium acnes* (formerly *Propionibacterium acnes*). Colonization and antibiotic resistance vary in different parts of the world; however, the *Staphylococcus aureus* and coliform strains have been considered more difficult to treat (Zimmerli et al., 2004; Grammatopoulos et al., 2017).

This study originally started as a part of our department's quality follow-up that evaluates all patients with an infected total hip arthroplasty (THA) treated within the NU Hospital Group in western Sweden (Norra Älvsborgs Community Hospital, which has an orthopedic emergency department, and Uddevalla Hospital, which has an elective care unit). This was in accordance with a national, interdisciplinary collaboration for safer prosthetic knee and hip operations (PRISS) that was presented in 2013 and suggested continuous local follow-up of routines and results (Lindgren et al., 2014). The purpose of our retrospective investigation was to evaluate all PJIs at a medium-sized Swedish hospital during a 7-year period with respect to infecting microorganisms, surgical treatment, antibiotics, and outcome.

## 2 Patients and methods

### 2.1 Design and data collection

Adult patients in the NU Hospital Group diagnosed with a PJI between 1 January 2012 and 31 December 2018 were included in the study. Information from three official database registers were collected. First the operative record's database of performed surgical interventions as well as the in-patient database in the NU Hospital Group were both searched for the ICD-10 code “T84.5”, indicating a deep periprosthetic joint infection. All discovered procedures were then controlled against our surgical data reported to and collected in the Swedish National Hip Registry. Our primary THAs between 2012 and 2014 were additionally matched with the Swedish Prescription Drug register in a follow-up study of infection registration based on the National Registry (Lindgren et al., 2014). The patient files for all registered infections were then collected and scrutinized by the authors to both confirm the diagnosis and match it according to the guidelines published by Musculoskeletal Infection Society (Parvizi et al., 2011) and the Infectious Diseases Society of America (Osmon et al., 2013) to ensure conformity. Patients with semi-total arthroplasties without an acetabular component were excluded, since these implants were only used in treatment of cervical hip fractures in old and fragile patients. As previously described these patients have considerable comorbidity, are often institutionalized, and both treatment and follow-up is unreliable (Guren et al., 2017).

### 2.2 Definitions

A PJI was considered present with the major criterium of two or more positive cultures with growth of the same microbiological organism in joint fluid or collected tissue samples. Additionally, at least two of the following criteria were present: wound discharge, sinuses, fever, local inflammation signs, raised CRP (> 10), or clinical findings of joint distress and particularly load pain.

Routinely, five tissue cultures were collected at implant exchange surgery from the interface between both the cup and the stem implant as well as cancellous bone when available, or otherwise including at least five samples from the joint capsule. The samples were harvested before perioperative intravenous antibiotic treatment was administrated. Antibiotic treatment was given according to established protocols (Osmon et al., 2013; Zimmerli et al., 2004) and national guidelines (Tevell et al., 2019) depending on antibiotic susceptibility and started with intravenous antibiotic therapy that was continued for 7–10 d followed by oral medication that was scheduled to terminate after 3 months.

The laboratory handling of microbiological tissues was changed in early 2015. Before, 10 samples were collected in broth and transported to the laboratory, where the material was plated on agar plates. From 2015, at least five tissue samples are routinely harvested at surgery and collected in empty sterile test tubes. Any further treatment of samples is performed in the laboratory. Transportation time is thereby considered less crucial, and the laboratory cultivates a fresh tissue section with imprint on agar substrate primarily. Afterwards, residual tissue pieces are continuously chopped for prolonged enrichment, which is routinely performed in all samples labeled “prosthesis”. This reduces the risk of rapidly and strongly growing species taking over and hiding slow-growing pathogens such as *Cutibacterium acnes*.

**Figure 1:**
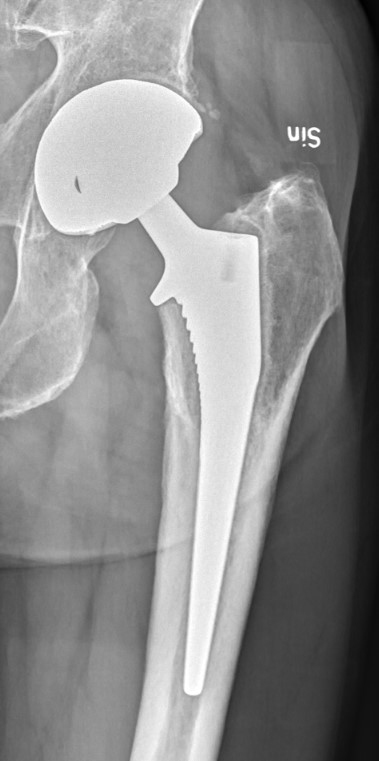
Radiograph of 4-year-old infection with coagulase negative staphylococci (CoNS) in uncemented THAs. Radiolucent lines are evident around the cup in Charnley zone 3 and around the stem in Gruen zones 1 and 7. © 2021 Urban Hedlundh.

An infection was classified as either postoperative or hematogenous. Cases with a sudden onset of inflammatory symptoms well beyond the postoperative period with a previously well-functioning arthroplasty were considered hematogenous (here ranging from 5 months to 25 years; median 6 years). In postoperative infections, we have refrained from distinguishing between acute and chronic PJI in accordance with the recommendation at the Second International Consensus Meeting on Musculoskeletal Infections (Elkins et al., 2019). In most of our postoperative PJIs there had been wound complications and a superficial site infection following the insertion of the implant without a continuous period with an optimally functioning joint. However, there were also infections with a probable origin in the prosthetic procedure with well healed surgical wounds and no septic episodes but a rehabilitation phase with continuous discomfort and loading pain. These infections were first evident after repeated visits, laboratory investigations, and finally progressive radiographic changes (Fig. 1).

A PJI was considered eradicated when there were no inflammatory signs including normalized ESR and/or CRP or symptoms from the hip joint for a minimum of 2 years after termination of antibiotic treatment, which is considered the shortest time in published literature to establish infection eradication (Sendi et al., 2017). Unrelated death (n=2) or hip revisions for other reasons than infection with negative perioperative cultures (n=3) occurring during follow-up within 2 years were labeled eradicated. Final failure was defined as an ongoing PJI with no planned future surgery but suppressive life-long antibiotic treatment. Girdlestone resection arthroplasties were reported separately since they were successful regarding infection eradication, but not from a prosthetic point of view.

**Table 1:** Surgical data on patients undergoing THA (primary and revision) at our institution between 2012 and 2018 compared with the infected THA patients operated on during the same period and included in the study. Please note that 18 of the study patients were primarily operated on elsewhere and 20 additional patients were operated on prior to 2012. Surgical risk was defined according to the American Society of Anesthesiologists physical status classification (ASA).

	THA	Infected THAs in study
	n=2975	n=51
Number of THAs		
Primary	2646	43
Revision	329	8
Gender		
Male	1270	32
Female	1705	19
Mean age (years ± SD)	69 ± 10	67 ± 14
Implant fixation		
Uncemented	1079	30
Cemented	1547	17
Hybrid	273	4
Missing	76	0
Mean body mass index (kg m${}^{-2}$, ± SD)	27.8 ± 4.9	30.0 ± 7.5
Missing data	265	4
Patient surgical risk		
ASA 1	488	8
ASA 2	1712	23
ASA 3	700	19
ASA 4	6	0
Missing data	69	1

### 2.3 Primary hip arthroplasty surgery

In all primary surgery from 2016, the patient arrived at the hospital on the day of the operation. Preoperative cleansing at home with sponges containing chlorhexidine gluconate (CHG) were advocated twice on the day before surgery as well as in the morning before leaving home. Prior to surgery the affected leg and groin was cleaned once more with CHG in the theater and then with chlorhexidine alcohol 50 % before draping. Between 15 and 30 min before skin incision, 2 g of flucloxacillin was administered intravenously followed by two additional doses 2 and 6 h after the first dose according to Swedish tradition and guidelines (Tevell et al., 2019). In 5 to 10 % of the patients another antibiotic was chosen due to allergy. The theater had laminar air flow and all persons in the room were dressed in reusable non-permeable polyester coveralls including helmets. The surgical staff used an additional reusable impenetrable gown and two pair of gloves.

### 2.4 Surgical grouping

**Figure 2:**
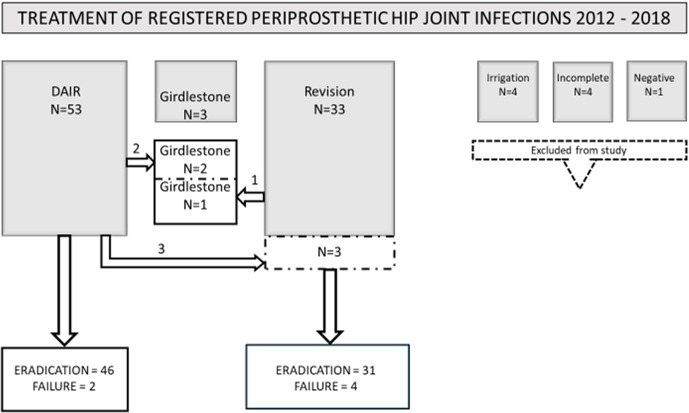
Presentation of all 89 total hip arthroplasties labeled periprosthetic joint infections (PJIs). Outcome of standardized treatment has been classified as “eradication” and “failure”.

All patients with at least two culture-positive tissue samples and/or joint aspiration with growth of the same microorganism were included in the study (Fig. 2). Four patients undergoing an outdated treatment with only drainage and limited irrigation due to high age and poor health status were excluded. In accordance with inclusion criteria, four patients who were diagnosed with a suspect joint infection by only one joint aspiration culture or a wound cultivation were excluded as well as one patient with a suspected but culture-negative PJI. The remaining 89 PJIs were divided in three groups according to surgical treatment (Fig. 2).

- *Debridement, antibiotics, and implant retention in accordance with published DAIR protocols (Tsang et al., 2017).* The joint was dislocated, and aggressive surgical debridement was performed by experienced revision surgeons followed by irrigation with 1–3 L of sterile sodium chloride before exchangeable modular heads and liners were changed. Resorbable, equine collagen fleeces containing between 70 and 280 mg of gentamicin were implanted in the joint before wound closure. The size of the fleece was usually not listed in the operative records. The procedure was planned to be performed within 6 weeks postoperatively but sometimes occurred later. Time from onset of symptoms was usually not specified in files since initial surgical wound care and agraffe removal was performed by district nurses and general practitioners. Hematogenous PJI was treated with DAIR within 2 weeks of presumed acute onset of symptoms.
- *One- or two-stage implant exchange (revision).* This was the preferred treatment in long-standing infections exceeding 6 months, less when radiographic zones were evident (Fig. 1), as well as when any loose implants were discovered during planned DAIR surgery. In 15 patients with loose stems but well-integrated cups in porous metal, there was only an exchange of liner, removal of cup screws, and implantation of gentamicin fleece in existing screw holes in addition to the stem exchange. Well integrated anchored titanium stems were left in four cases, and here only the cup and modular head was changed. All cemented components were revised, and adjacent detectable bone cement removed.
- *Complete and permanent removal of implants in a Girdlestone-like procedure (Vincenten et al., 2019).*

### 2.5 Statistics

Descriptive statistics were used to summarize demographic data, time, antibiotic treatment, and involved microorganisms. Calculations of group comparisons using 2×2 contingency tables were performed by Fisher's exact test. Non-parametric group comparisons used the Mann–Whitney U test. A p value of < 0.05 was considered statistically significant.

**Table 2:** Isolated microorganisms in 89 revised periprosthetic hip joint infections. The number of cultures was 110 since significant growth (≥ two tissue samples) of more than one bacterium was noted in 21 patients. Cultures in patients with repeated surgery and the same bacterial strains have only been counted once.

		Monomicrobial	Eradication			
Organism	n (%)	infection	n ( %)	Failure	Girdlestone	$p^{\text{c}}$
Gram-positive bacteria						
CoNS${}^{\text{a}}$	33 (30)	23	30 (91)	2	1	0.27
*Staphylococcus aureus*	22 (20)	16	12 (55)	6	4	0.0001
*Cutibacterium acnes*	18 (16)	14	18 (100)	0		0.07
*Streptococcus* spp.	15 (14)	8	14 (93)	1		0.46
*Enterococcus* spp.	8 (7)	2	7 (88)	1		1.0
*Corynebacterium* spp.	2 (2)	0	2 (100)	0		1.0
Gram-negative bacteria						
*Escherichia coli*	5 (5)	4	4 (80)	0	1	0.58
*Enterobacter* spp.	2 (2)	1	2 (100)	0		1.0
Other${}^{\text{b}}$	5 (5)	0	4 (80)	1		0.58

${}^{\text{a}}$ CoNS = coagulase negative staphylococci. ${}^{\text{b}}$ *Hemophilus influenzae, Klebsiella oxytoca, Serratia* sp., *Acinetobacter* sp., *Eikenella corrodens*. ${}^{\text{c}}$ Calculated on treatment failure including Girdlestone vs. eradication in the specific bacterium/group of bacteria vs. all other.

## 3 Results

### 3.1 Study population, patient characteristics, and survival

A total of 87 patients with 89 PJI episodes were included in the study. One patient with bilateral arthroplasties had a simultaneous bacterial precipitation in both hips and bilateral DAIR treatment, while another was initially successfully treated but infected with a new pathogen after further revision surgery. A total of 18 primary arthroplasties had been performed in other hospitals at a median of 2 years (23 d–24 years) before the patients attended care at our institution. Median age at the time of diagnosis was 68 years (40–92); 31 of the patients were female (34 %), and 12 patients died from causes not related to the PJI at an average of 59 months (16–90) after revision surgery and excision arthroplasties. No deaths directly attributable to the PJI were identified.

### 3.2 Microbiology

Distribution of bacteria in collected tissue samples is presented in Table 2.

Coagulase negative staphylococci (CoNS), *Staphylococcus aureus*, and *Cutibacterium acnes* were the three most prevalent bacteria and were detected in 30 %, 20 %, and 16 % of the cultures, respectively. Multiple organisms were found in 24 % of the cases while 76 % of the infections were monomicrobial with no difference in failure rate (2 out of 19 vs. 10 out of 58; p=0.7). Final surgical failure resulting in life-long antibiotic suppression (n=6) or Girdlestone (n=6) was highly associated with *Staphylococcus aureus* infection (10 out of 12; 83 %; p=0.0001) (Table 2).

*Cutibacterium acnes* was isolated in 18 of 89 PJIs (20 %), in 14 out of 18 cases (78 %) as the single growing pathogen. A total of 15 cases (83 %) were diagnosed after 2015. Patient characteristics in *Cutibacterium* infections compared with other infectious agents are presented in Table 3. DAIR (n=11) or revision implant exchange (n=7) was successful in all cases. *Cutibacterium acnes* infections were seen in 15 out of 18 of the uncemented implants (83 %) as compared to 30 out of 71 (42 %) of all other PJIs (P=0.003) (Table 3).

**Table 3:** Differences between periprosthetic joint infections with growth of *Cutibacterium acnes* in tissue samples and other bacteria in 89 patients with total hip arthroplasty.

	*Cutibacterium acnes*	Other bacteria	
	n=18	n=71	p
Gender			
Male	13	45	
Female	5	26	0.59
Median age, years (range)	65 (40–75)	70 (46–92)	0.20
Body mass index			
Median BMI, kg m${}^{-2}$ (range)	33.8 (24.1–45.0)	28.3 (20.1–48.4)	0.16
Missing data	0	15	
Surgical risk			
ASA 1	3	8	
ASA 2	10	31	
ASA 3	5	26	
ASA 4	0	1	0.35${}^{\text{a}}$
Missing data	0	5	
Primary implant fixation			
Uncemented	15	30	
Cemented/hybrid	3	41	0.003
Polymicrobial influence			
Single microbe	14	54	
Polymicrobial	4	17	1.00
Result surgical treatment			
Eradication	18	59	
Failure	0	6	
Girdlestone	0	6	0.12${}^{\text{b}}$

ASA: American Society of Anesthesiologists physical status classification. ${}^{\text{a}}$ Calculated on ASA 1 + ASA 2 vs. ASA 3 + ASA 4. ${}^{\text{b}}$ Calculated on treatment failure including Girdlestone vs. eradication in patients with growth of *Cutibacterium acnes* vs. all other.

### 3.3 Treatment and outcome

The flow chart for the patients in the study is summarized in Fig. 2. Treatment with DAIR was performed in 53 out of 89 PJI episodes (60 %). Failure was noted in 7 (13 %) of these procedures. Two chronic failures ended up with retained prostheses and lifelong antibiotic suppression; three of the patients suffered extended implant exchanges (revisions) and two Girdlestones which all resulted in infection eradication. Two patients with wound complications after DAIR were treated with vacuum-assisted closure (VAC) and received antibiotics for an additional 3 and 8 months, respectively. There was no sign of infection recurrence 2 and 3 years after antibiotic termination.

A total of 36 primary revision implant exchanges were performed, resulting in 28 out of 36 (78 %) one-stage revisions and 8 out of 36 (22 %) two-stage revisions. The outcome of all implant exchange surgery is presented in Table 4. Peri- and postoperative antibiotic treatment was given in accordance with preoperative aspiration and wound cultures when available. Follow-up peroral antibiotics were given following the results of tissue cultures and in collaboration with an infectious diseases specialist. Changes of antibiotic therapy were made in approximately 5 % of patients according to allergy or other toxic effects.

A total of 34 infections (38 %) were classified as hematogenous while 55 (62 %) were postoperative. The postoperative infections also included three failed DAIR treatments (5 %) which were later successfully revised with infection eradication. A statistically inferior final outcome of hematogenously infected patients was not proven (8 out of 34 vs. 4 out of 55; p=0.052).

**Table 4:** Summary of revision procedures in hematogenous and postoperative periprosthetic joint infections.

	n (%)
Type of revision surgery	
DAIR	53
Eradication	46 (87)
Revision	3
Failure	2
Girdlestone	2
Revision implant exchange	36
One-stage	28 (78)
Eradication	26 (93)
Failure	2
Girdlestone	0
Two-stage	8 (22)
Eradication	5 (63)
Failure	2
Girdlestone	1
Immediate Girdlestone	3
Primary antibiotic treatment	
Dicloxacillin	20 (24)
Clindamycin	10 (12)
Cefotaxime	10 (12)
Vancomycin	33 (38)
Piperacillin/tazobactam	13 (15)
Other/missing	3
Type of infection	
Hematogenous	34 (38)
Post-operative	55 (62)
Outcome of hematogenous PJI	
Eradication	26 (76)
Failure	4
Girdlestone	4
Outcome of post-operative PJI	
Eradication	51 (93)
Failure	2
Girdlestone	2

DAIR: debridement and retention of implant; PJI: prosthetic joint infection.

The time of occurrence of the postoperative infections is presented in Table 5. In patients with revision surgery (DAIR or implant exchange, excluding Girdlestones) performed within 30 d after THA, infection eradication was seen in 88 % (21 out of 24) as compared to 95 % (20 out of 21) in patients revised between 31 and 180 d after primary surgery. Among patients with a later time point for revision (> 180 d, n=12) three patients had previously undergone a DAIR. The causative pathogens in treatment failures of postoperative infections within 180 d (n=4) including one primary Girdlestone were monobacterial CoNS in two cases and *Staphylococcus aureus* and *Enterococci* in one case each respectively.

**Table 5:** Distribution and outcome of postoperative infections during different time intervals after total hip arthroplasty (THA). Day 0 = time point for THA. Failed DAIR in 3 patients resulted in 58 treatment procedures in 55 patients.

	Total	DAIR	Implant	Girdlestone	Eradication	Failure	Median day of
	n	n	exchange n	n	n (%)	n	revision surgery
Day 1–30	24	21	2	1	21 (88)	2	24
Day 31–180	21	18	3	0	20 (95)	1	53
Day 181–	13	3	9	1	10 (77)	2	554

DAIR = debridement and implant retention.

## 4 Discussion

We investigated 89 infected THAs in 87 patients during a period of 7 years regarding bacterial findings and treatment outcome with respect to different surgical methods. The number of PJI episodes was relatively small and, like in other observational studies, this creates a risk that the diversity of both the patient populations and the treatment strategies cause inconsistent recommendations despite our best efforts. Nevertheless, our results support recently published treatment algorithms regarding PJI (Born et al., 2016; Grammatopoulos et al., 2017; Sendi et al., 2017) and are comparable with previously presented meta-analysis data concerning revisions (Leonard et al., 2014) and DAIR (Tsang et al., 2017). The data also point at a possible relationship between infections with *Cutibacterium acnes* and uncemented hip implants.

With the exception of *Cutibacterium acnes*, the isolated bacterial agents in our study cohort did not differ largely from other reports on prosthetic joint infections with a majority of infections caused by staphylococci (Zimmerli et al., 2004; Grammatopoulos et al., 2017). Multiresistant organisms are, however, less common in Sweden than in many other countries, probably due to a more restrictive use of broad-spectrum antibiotics, which also justifies the use of Flucloxacillin as the principal preoperative prophylaxis (Molstad et al., 2008). A majority of the failures in our study cohort (83 %) were associated with PJIs caused by *Staphylococcus aureus*, which is known as a potent biofilm producer and is often difficult to eradicate (Moormeier and Bayles, 2017; Sendi et al., 2017).

We found an unexpectedly high number of *Cutibacterium acnes* infections among our patients. In fact, this was the third dominating species occurring in 16 % of the patients. Improved microbiological analyses during the study period may have contributed to these findings since 15 out of 18 cases (83 %) were diagnosed after the introduction of a new method of analysis at the local laboratory in 2015. The role of *Cutibacterium acnes* as a true pathogen and not a commensal in PJIs has been discussed (Lavergne et al., 2017). Nevertheless, in all our cases the bacterium was found in at least two tissue specimens and directed treatment was successful, which would support its causative role in these infectious episodes. We did not confirm any significant differences regarding age, sex, BMI, or ASA class when comparing patients with PJIs caused by *Cutibacterium acnes* and other pathogens. However, a small-sized study like ours may be underpowered regarding lower median age and a higher incidence of male patients, which has previously been described in *Cutibacterium acnes* infections (Levy et al., 2008; Mook et al., 2015).

*Cutibacterium acnes* has mainly been reported in open shoulder surgery and shoulder arthroplasty (Levy et al., 2008; Mook et al., 2015). The vicinity to the upper thorax with presence of acne vulgaris and to the axilla with lipid-rich sebaceous hair follicles has been designated as the cause, since the bacterium is 10 times more common in this region compared with lower limb infections (Levy et al., 2008). However, our results indicate that *Cutibacterium acnes* infections in other joint prostheses may be underdiagnosed. Whether this should cause changes in the antibiotic prophylaxis of certain patient groups requires significantly larger studies.

The relationship between infections with *Cutibacterium acnes* and uncemented hip implants in our study is striking, particularly considering that only one-third of the total implants during the study period were uncemented. Nevertheless, this is significantly more than the 21 % in Sweden during the corresponding period reported by the National Register. This might, if the observation of a relationship between *Cutibacterium acnes* and uncemented hip implants is correct, be a reason for our unusually high proportion of *Cutibacterium acnes* infections. In cemented THAs we have been using the Optipac Refobacin Bone Cement R system (ZimmerBiomet, Dieticon, Switzerland) with the 40 g package for cup fixation and 60 g mixed for stem fixation. In the 40 g of the cement polymer is 0.5 g of active gentamicin, which has an established bactericidal effect on particularly gram-positive organisms like *Staphylococcus aureus* and CoNS (Kendoff et al., 2016; Khassebaf et al., 2015). *Cutibacterium acnes* growing in biofilm has proven susceptibility to locally administered gentamicin (Ramage et al., 2003). It might thus be reasonable to assume an effect on the bacteria by the release of antibiotics from the cement rather than by the influence of different alloys and coatings of the implants (Lenguerrand et al., 2018; Braem et al., 2014).

The short-term clinical outcome of the *Cutibacterium* infections was successful. Recommended treatment with benzylpenicillin followed by amoxicillin or clindamycin perorally in single therapy or in combination for 3 months together with adequate surgery (Boisrenoult, 2018) resulted in infection eradication in all cases. The Addition of rifampicin with its more serious side effects does not seem to offer any benefits (Jacobs et al., 2016) and was in this cohort used only in a few polymicrobial infections.

Our high proportion of one-stage revisions (78 %) differs from the Swedish and international tradition but has not shown any negative impact on infection eradication with more than 90 % successful one-stage revisions. This is also in agreement with another recent Swedish study by Svensson et al. (2019) based on data from the Swedish Hip Arthroplasty Register.

Establishment of mature biofilm is undoubtedly related to the length of time during which bacteria affect the implant. However, the optimal interval between THA and the performance of DAIR in postoperative cases has not been established. Sendi and coworkers suggested an interval of ≤ 30 d after primary or revision hip arthroplasty (Sendi et al., 2017). In a large proportion of our cases the time from apparent infection to surgical intervention was substantially longer, as seen in Table 5, with an average of 45 d after THA surgery. Apparent reasons for this were that initial surgical wound care and agraffe removal was performed by district nurses and general practitioners before the patient attended care in the emergency unit. Here, joint aspiration was performed by educated ultrasound radiologists. Referral to a competent hip revision surgeon took place about a week later, surgery after another 2–8 d. Although the shortest possible time to surgery in postoperative infections is always desirable, we prioritized surgical experience and consulting the appropriate specialist in infectious diseases. Despite the delayed surgical intervention, the treatment was successful in more than 90 % of the patients that were operated on between 31 and 180 days after THA.

The prognosis regarding eradication of a PJI is certainly affected by a combination of factors including the causative microorganism, its ability to form biofilm, type of implant, surgical radicality, and duration of infection. Our material was too small to allow for comparisons between different pathogens in this aspect. The failures among our post-operative infections could also not be related to any specific pathogen. In addition, the sample size was not large enough to establish a statistical significance regarding the outcome of hematogenous vs. post-operative infections.

A major limitation of this study is its retrospective nature reflecting the care at one medium-sized regional hospital. The limited number of patients and the heterogenous series renders difficulties in comparisons and a risk of type-one error. On the other hand, we have possibly included all infected THAs during a relatively long time period. All PJIs were verified by two or more positive tissue cultures with growth of the same pathogen. Surgery was performed by a few experienced surgeons with similar technique and antibiotic therapy following established guidelines.

Optimal treatment of PJI regarding specific infectious agents when it comes to combining surgery and antibiotics requires large multicenter studies that are preferably prospectively randomized. Investigations of the correlation between certain types of implants and bacterial colonization should be made possible by cross-checking results from national registers and bacteriological data banks. Our study might in this aspect serve as a pilot study with new implications.

## 5 Conclusions

We found an unexpectedly high number of *Cutibacterium acnes* infections, which raises questions as to the choice of antibiotic prophylaxis. Our results indicate that the use of uncemented implants may be a risk factor for PJIs caused by *Cutibacterium acnes*. Although post-operative infections are preferably surgically treated with DAIR within a month after hip replacement, infection eradication may still not be excluded at an extended time provided there are certain types of bacteria, assured implants, targeted antibiotic selection, and radical surgery by experienced surgeons. Further large-scale studies are needed to confirm our results.

## Data Availability

Data are presented in the tables and figures. Depersonalized individual patient data are provided for reviewers, but not publicly publishable for ethical reasons, since individual patients can be identified.
